# Supplementary material for: HIV-1 CD4-binding site germline antibody–Env structures inform vaccine design
Source: Nat Commun. 2022 Oct 17;13:6123. doi: 10.1038/s41467-022-33860-2 (PMC9576718; doi:10.1038/s41467-022-33860-2)
Supplement: Supplementary file 1 — Supplementary Information [file 41467_2022_33860_MOESM1_ESM.pdf]

Supplementary Information

HIV-1 CD4-binding site germline antibody–Env structures inform vaccine design

Kim-Marie A. Dam[1], Christopher O. Barnes[1‡], Harry B. Gristick[1], Till Schoofs[2,3,4§], Priyanthi N.P. Gnanapragasam[1], Michel C. Nussenzweig[2,5], Pamela J. Bjorkman[1]

[1]Division of Biology and Biological Engineering, California Institute of Technology, Pasadena, CA, USA

[2]Laboratory of Molecular Immunology, The Rockefeller University, New York, NY, USA

[3]Laboratory of Experimental Immunology, Institute of Virology, University of Cologne, Faculty of Medicine and University Hospital of Cologne, Cologne, Germany

[4]German Center for Infection Research, Partner Site Bonn–Cologne, Cologne, Germany

[5]Howard Hughes Medical Institute, Chevy Chase, MD, USA

[‡]Present address: Department of Biology, Stanford University, Stanford, CA, USA

[§]Present address: GlaxoSmithKline Vaccines, 1330 Rixensart, Belgium

Corresponding author: Pamela Bjorkman

Email: bjorkman@caltech.edu

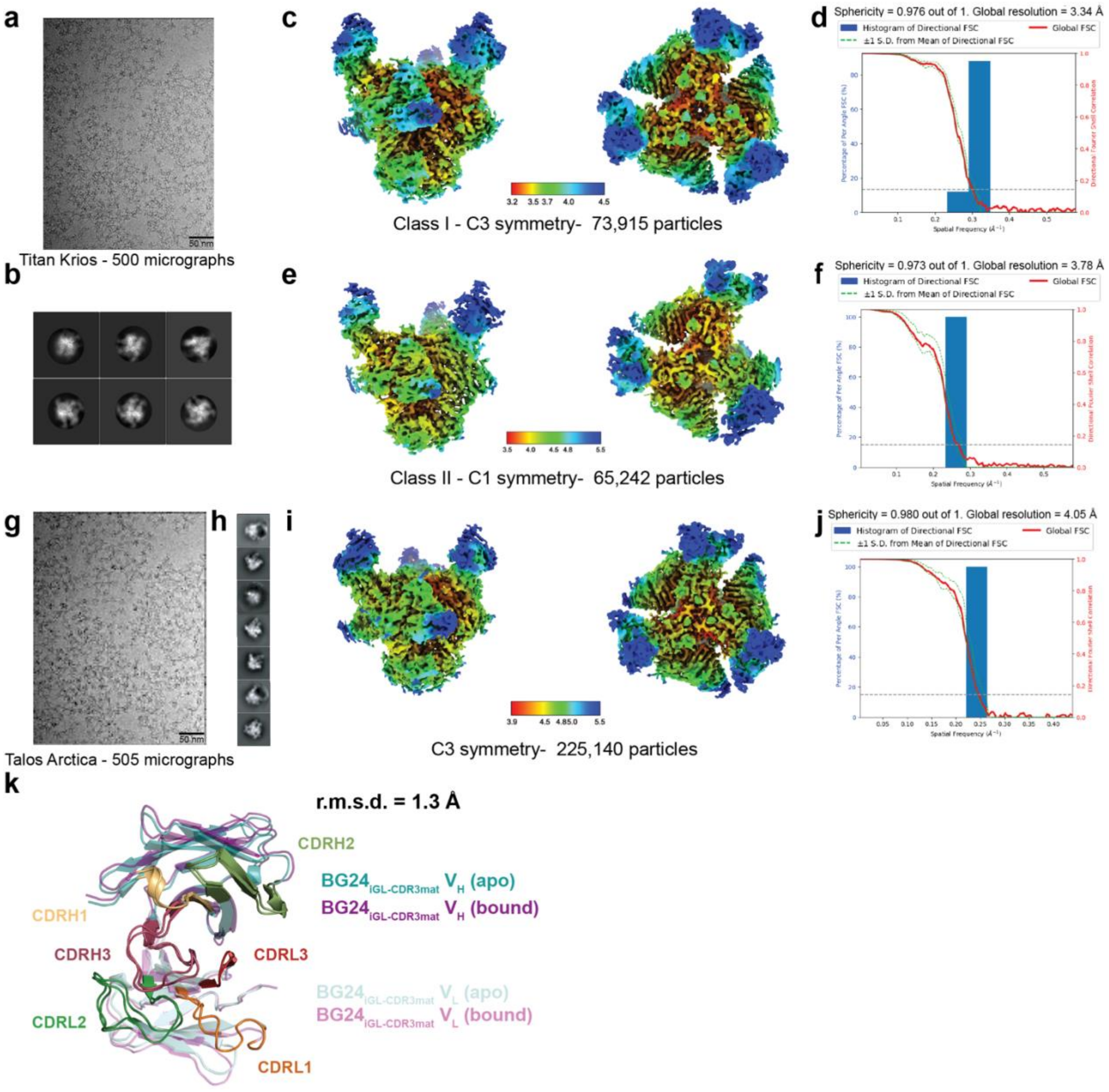

Supplementary Fig. 1 | Cryo-EM data processing and validation for BG24$_{iGL}$- GT1-10-1074 complexes and BG24$_{iGL\text{-}CDR3mat}$ Fab alignment

a, Representative micrograph and **b**, cryo-EM 2D class averages for the BG24$_{iGL}$-$_{CDR3iGL}$- GT1-10-1074 cryo-EM structures. For this dataset, two classes were resolved: Class I with three BG24$_{iGL\text{-}CDR3iGL}$ Fabs bound to GT1, and Class II with two BG24$_{iGL\text{-}CDR3iGL}$ Fabs bound to GT1. Differences in the cryo-EM density map for Class I and Class II of the BG24$_{iGL}$-$_{CDR3iGL}$-GT1-10-1074 complex were not observed. The **c**, local resolution map and **d**, gold-standard Fourier

shell correlation (FSC) plots for $BG24_{iGL}$-$_{CDR3iGL}$- GT1-10-1074 Class I. The **e**, local resolution map and **f**, gold-standard Fourier shell correlation (FSC) plots for $BG24_{iGL}$-$_{CDR3iGL}$- GT1-10-1074 Class II. **g**, Representative micrograph, **h**, cryo-EM 2D class averages, **i**, local resolution estimations, and **j**, gold-standard Fourier shell correlation (FSC) plots for $BG24_{iGL\text{-}CDR3mat}$-GT1-10-1074. **k**, Alignment and r.m.s.d. (Å) of the crystal structure of apo $BG24_{iGL\text{-}CDR3mat}$ Fab and the $BG24_{iGL\text{-}CDR3mat}$ Fab bound to GT1 in the cryo-EM structure.

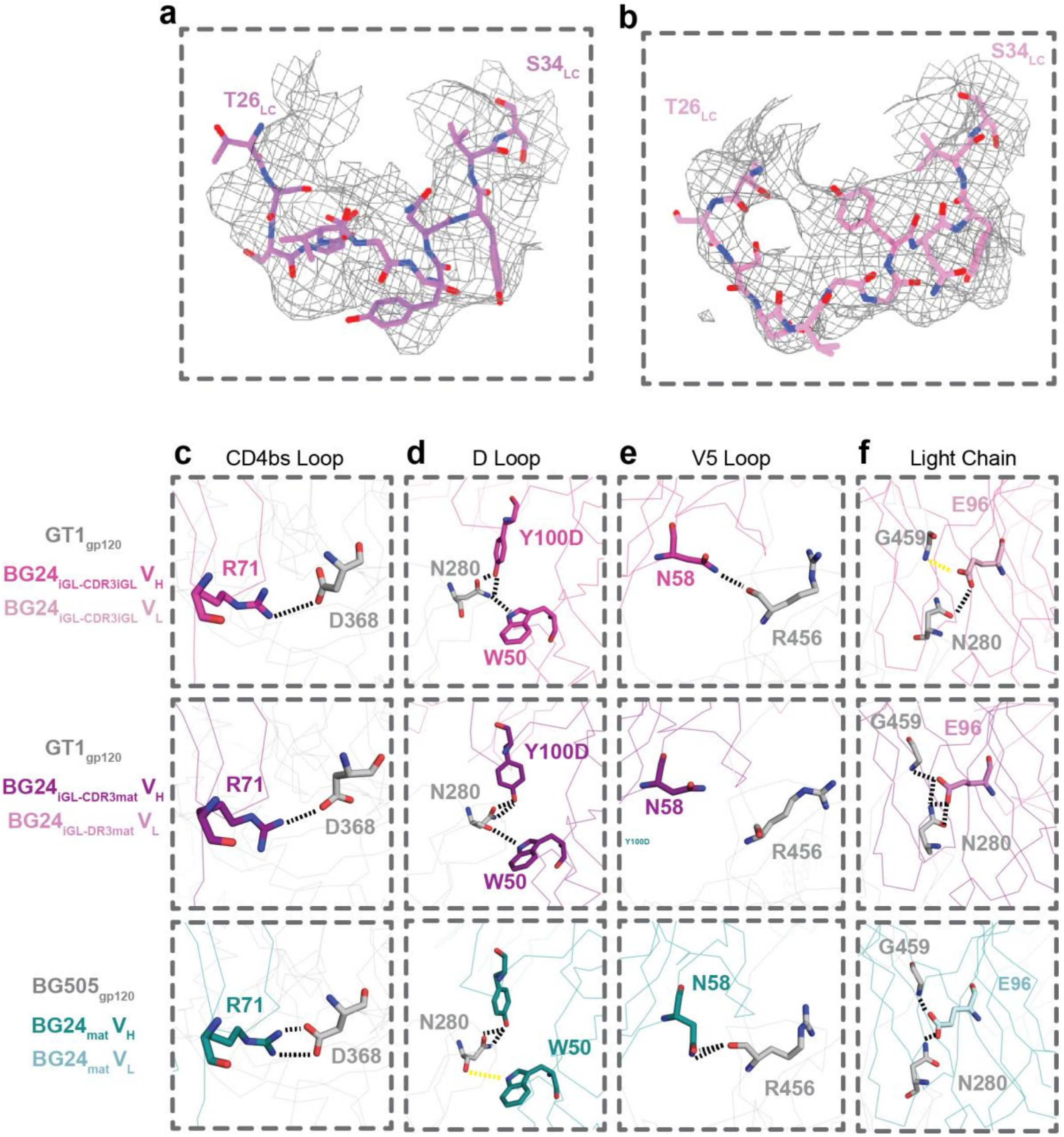

Supplementary Fig. 2 | BG24$_{iGL}$ CDRL1 local density and VH1-2*02 signature contacts comparison among BG24$_{iGL}$ and BG24$_{mat}$ structures

Local density for CDRL1 with modeled LC residues T26-S34$_{LC}$ (stick representation) for **a**, BG24$_{iGL-CDR3mat}$ and **b**, BG24$_{iGL-CDR3iGL}$ contoured to 3.8 and 3.6 σ, respectively. Interactions in **c**, CD4bs, **d**, D Loop, **e**, V5 loop, and **f**, LC for BG24$_{iGL-CDR3iGL}$-GT1$_{gp120}$, BG24$_{iGL-CDR3mat}$-GT1$_{gp120}$,

and $BG24_{mat}$-$BG505_{gp120}$ structures. Contacts between atoms within 4 Å are represented by black dotted lines; contacts between 4-5 Å are represented by yellow dotted lines.

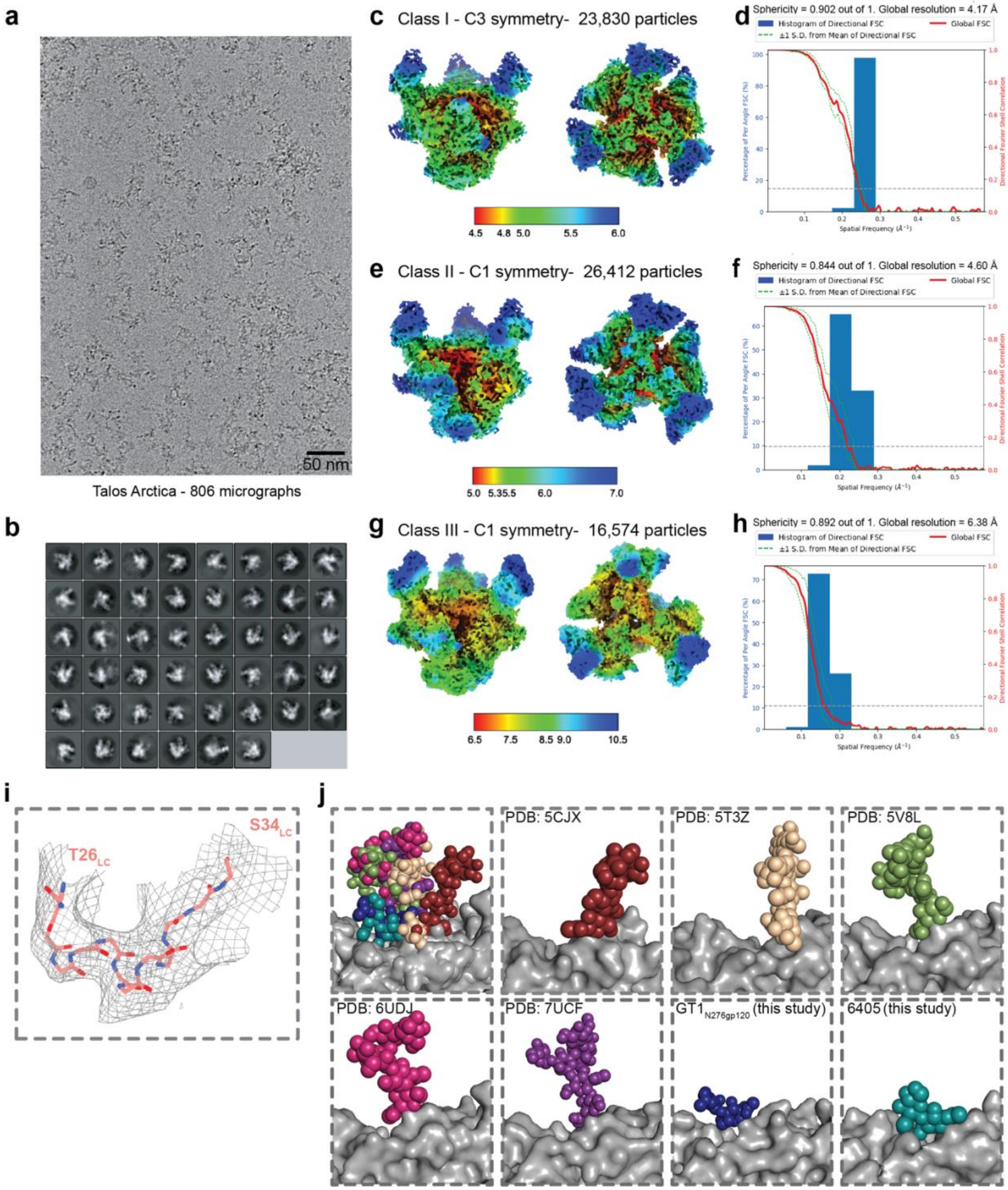

Supplementary Fig. 3 | Cryo-EM data processing and validation for BG24$_{LC\text{-}iGL}$-GT1$_{N276gp120}$-10-1074 complex, BG24$_{LC\text{-}iGL}$ CDRL1 local density, and analysis of N276$_{gp120}$ glycan flexibility

a, Representative micrograph, **b**, cryo-EM 2D class averages for the $BG24_{LC\text{-}iGL}$- $GT1_{N276gp120}$-10-1074 cryo-EM structures. For this dataset, three classes were resolved: Class I with three $BG24_{LC\text{-}iGL}$ Fabs bound to $GT1_{N276gp120}$, Class II with two $BG24_{LC\text{-}iGL}$ Fabs bound to $GT1_{N276gp120}$, and Class III with one $BG24_{LC\text{-}iGL}$ Fab bound to $GT1_{N276gp120}$. The **c**, local resolution map and **d**, gold-standard Fourier shell correlation (FSC) plots for $BG24_{LC\text{-}iGL}$- $GT1_{N276gp120}$-10-1074 Class I. The **e**, local resolution map and **f**, gold-standard Fourier shell correlation (FSC) plots for $BG24_{LC\text{-}iGL}$- $GT1_{N276gp120}$-10-1074 Class II. The **g**, local resolution map and **h**, gold-standard Fourier shell correlation (FSC) plots for $BG24_{LC\text{-}iGL}$- $GT1_{N276gp120}$-10-1074 Class III. **i**, Local density for CDRL1 with modeled backbone for LC residues T26-$S34_{LC}$ (stick representation) for $BG24_{LC\text{-}iGL}$ contoured to 5.4 σ. **j**, Comparison and overlay of the $N276_{gp120}$ glycan from existing Env structures and $GT1_{N276gp120}$ and 6405 from this study.

```
Consensus XNLWVTVYYGVPVWKDAETTLFCASDAKAYETEKHNVWATHACVPTDPNPQEIHLENVTEEFNMWKNNMVEQMHTDIISL
BG505     S...............................................................................
GT1       E...............................K...............................................
6405      ND.............E.K..........S....A..I...............VE.V....N......D..D...E.....

Consensus WDQSLKPCVKLTPLCVTLQCTNVTNNITDD-----------MRGELKNCSFNMTTELRDKXQKVHALFYRLDIVPINENQ
BG505     ..............................-----------...................K...YS......V.Q.....
GT1       ..............................-----------...................R........K..........
6405      ..E.................SEMK.GTRGASNSTGTNNGTISSTDRM.....A..VVN..QK...........E..GN--

                         197                                 234
Consensus ------XNTEYRLINCNTSAITQACPKVSFEPIPIHYCAPAGFAILKCKDKKFNGTGPCPSVSTVQCTHGIKPVVSTQLL
BG505     GNRSNNS.K.......................................................................
GT1       -------..S........A.............................................................
6405      ------NS...M.........A.S...IT...............L...R..Q.......TN..S................

                 Loop D
                 276
Consensus LNGSLAEEEVMIRSENITNNAKNILVQFNTPVQINCTRPNNNTRKSIRIGPGQAFYATG--DIIGDIRQAHCNVSKATWN
BG505     ...........................................................--...................
GT1       ...............D.R...................................W.....--...................
6405      ........DII.....L.D.T.T.I..L.KSIA......Y....QR.H......IWTNDARV.V...........RVD..

                                   CD4bs Loop                           V4 Loop      β20/β21
                 355     363                         386   392
Consensus ETLGKVVKQLRKHFGNNTIIRFANSSGGDLEVTTHSFNCGGEFFYCNTSGLFNSTWISNTSVQGSNSTGSNDSITLPCRI
BG505     ................................................................................
GT1       ..............................................D.................................
6405      NMTQWAATK.GSLYN-RST.I.NHA....P.I.....T..................DIMN----TSPNNTDPI...Q...

               β20/β21                      β23  V5 Loop  β24
                                                  461 465
Consensus KQIINMWQRIGQAMYAPPIQGVIXCXSNITGLILTRDGG-STXSTTETFRPGGGDMRDNWRSELYKYKVVKIEPLGVAPT
BG505     .......................R.V.............-..N.....................................
GT1       .......................WMCQQYHR........-..D........S............................
6405      ........GV.R.I.....A.Q.L.S......L......ADNS.HN........N.K...................I...

Consensus RCKRRVVG
BG505     ........
GT1       ........
6405      KAR....E
```

Supplementary Fig. 4 | Sequence Alignment of BG505, GT1, and 6405 gp120

The sequence alignment for BG505, GT1, and 6405 gp120 residues and assigned consensus sequence. PNGS sequons in the consensus sequence are boxed in red, with residues numbers corresponding to Asn in the PNGS indicated above the sequence. PNGSs that are not conserved are highlighted in yellow, again with residues numbers corresponding to the Asn in the PNGS indicated above the sequence.

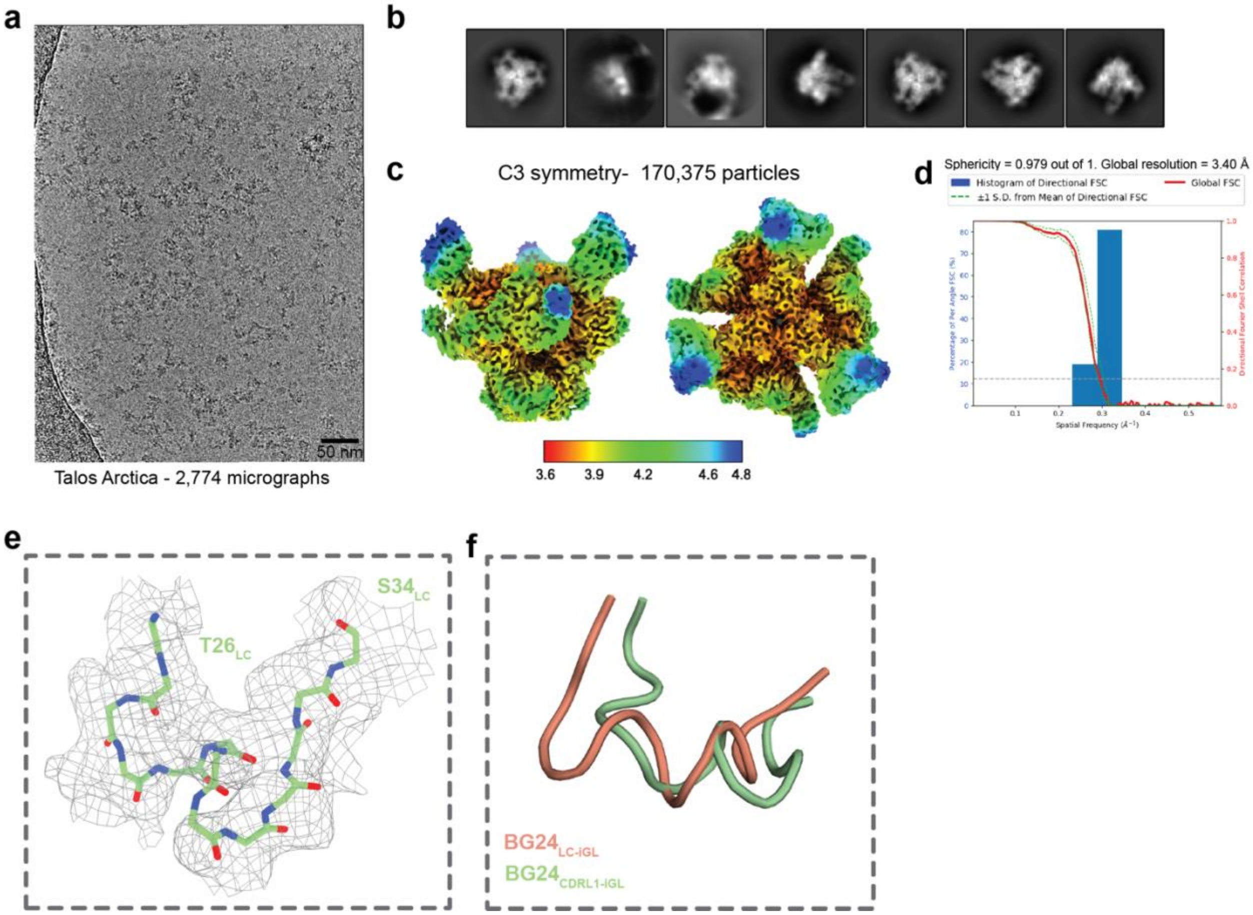

Supplementary Fig. 5 | Cryo-EM data processing and validation for BG24$_{CDRL1\text{-}iGL}$-6405-10-1074 complex and BG24$_{CDRL1\text{-}iGL}$ CDRL1 local density

a, Representative micrograph, **b**, cryo-EM 2D class averages, **c**, local resolution estimations, and **d**, gold-standard Fourier shell correlation (FSC) plots for BG24$_{CDRL1\text{-}iGL}$-6405-10-1074. **e**, Local density for CDRL1 with modeled backbone for residues T26-S34$_{LC}$ (stick representation) for BG24$_{CDRL1\text{-}iGL}$ contoured to 4.4 σ. **f**, Alignment of BG24$_{iGL}$ CDLR1s from the BG24$_{LC\text{-}iGL}$-GT1$_{N276gp120}$-10-1074 structure and from the BG24$_{CDRL1\text{-}iGL}$-6405-BG24$_{LC\text{-}iGL}$-10-1074 structure. CDRL1s are represented in cartoon.

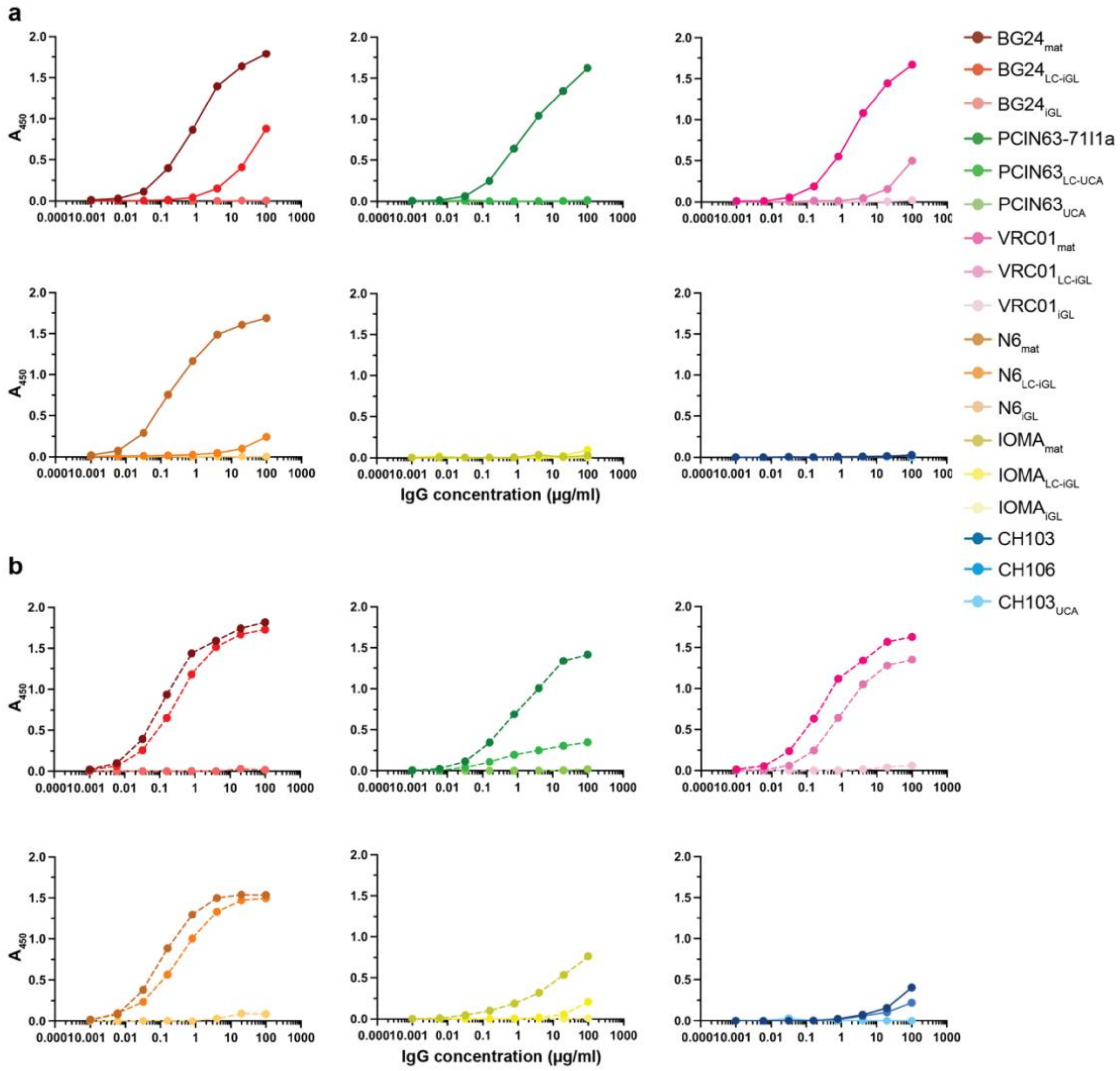

Supplementary Fig. 6 | ELISA curves of CD4bs IgGs bound to 6405 and $6405_{delN276gp120}$

ELISA curves corresponding to CD4bs IgGs bound to **a**, 6405 and **b,** $6405_{delN276gp120}$ SOSIPs.

Values are shown as mean ± s.d. of two individual biological replicates (n=2).

Supplementary table 1. Cryo-EM data collection, refinement, and validation statistics

	$BG24_{iGL-CDR3iGL}$- GT1-10-1074	$BG24_{iGL-CDR3mat}$- GT1-10-1074	$BG24_{LC-iGL}$- $GT1_{N276gp120}$-10-1074	$BG24_{CDRL1-iGL}$-6405-10-1074
EMDB:	EMD-26490	EMD-26492	EMD-26493	EMD-26496
PDB:	7UGN	7UGO	7UGP	7UGQ
**Data collection and processing**				
Magnification*	105,000x	73,000x	45,000x	45,000x
Voltage (kV)	300	200	200	200
Electron exposure (e–/Å^2)	60	60	60	60
Defocus range (μm)	1.2-3.0	1.2-3.0	1.2-3.0	1.2-3.0
Pixel size (Å)	0.8654	1.436	0.869	0.869
Recording mode	Counting	Counting	Counting	Counting
Symmetry imposed	C3	C3	C3	C3
Initial particle images (no.)	139,157	422,161	178,814	770,375
Final particle images (no.)	73,915	225,140	23,830	170,897
Overall map resolution (Å) (masked/unmasked)**	3.4 (3.7)	4.1 (5.0)	4.2 (4.8)	3.4 (3.7)
**Refinement**				
Initial model used (PDB code)	5T3Z	5T3Z	5T3Z	5T3Z
Map and model CC	0.84	0.82	0.73	0.79
Map sharpening *B* factor (Å^2)	-77	-150	-138	-119
Model composition				
Protein residues	3150	3138	3111	3162
Glycan residues	67	87	66	135
Validation				
MolProbity score	1.64	1.72	1.97	2.08
Clashscore	5.95	5.97	11.63	13.18
Poor rotamers (%)	0.04	0	0.11	0.26
Ramachandran plot				
Favored (%)	95.5	94.2	94.3	92.9
Allowed (%)	4.5	5.8	5.7	7.1
Disallowed (%)	0	0	0	0
RMS deviations				
Length (Å)	0.002	0.002	0.002	0.004
Angles (°)	0.508	0.491	0.539	0.606

* Nominal magnification; ** FSC threshold 0.143

Supplementary table 2. X-ray data collection and refinement statistics (molecular replacement)

PDB ID	iGL BG24 Fab *(12-2, SSRL)* 7UGM
**Data collection**[a]	
Space group	$P2_12_12_1$
Unit cell (Å)	53.2, 70.8, 134.9
α, β, γ (°)	90, 90, 90
Wavelength (Å)	1.0
Resolution (Å)	38-1.4 (1.42-1.4)
Unique Reflections	101,139 (49,320)
Completeness (%)	100 (99.9)
Redundancy	19.7 (19.2)
$CC_{1/2}$ (%)	99.3 (67.2)
<I/σI>	11.0 (1.2)
Mosaicity (˚)	0.17
R_{merge} (%)	16 (103)
R_{pim} (%)	3.7 (237)
Wilson *B*-factor	14.2
**Refinement and Validation**	
Resolution (Å)	38-1.4
Number of atoms	
Protein	3,189
Water	447
R_{work}/R_{free} (%)	19.3/20.8
R.m.s. deviations	
Bond lengths (Å)	0.006
Bond angles (˚)	0.93
MolProbity score	1.42
Clashscore (all atom)	4.6
Poor rotamers (%)	0.3
Ramachandran plot	
Favored (%)	96.9
Allowed (%)	3.1
Disallowed (%)	0.2
Average *B*-factor (Å)	
Protein	24.3
Water	32.7
