# Supplementary material for: HIV-1 CD4-binding site germline antibody–Env structures inform vaccine design
Source: Nat Commun. 2022 Oct 17;13:6123. doi: 10.1038/s41467-022-33860-2 (PMC9576718; doi:10.1038/s41467-022-33860-2)
Supplement: Supplementary file 2 — Reporting Summary [file 41467_2022_33860_MOESM2_ESM.pdf]

nature portfolio

Corresponding author(s): Pamela J. Bjorkman

Last updated by author(s): Sep 21, 2022

Reporting Summary

Nature Portfolio wishes to improve the reproducibility of the work that we publish. This form provides structure for consistency and transparency in reporting. For further information on Nature Portfolio policies, see our Editorial Policies and the Editorial Policy Checklist.

Statistics

For all statistical analyses, confirm that the following items are present in the figure legend, table legend, main text, or Methods section.

n/a	Confirmed	
☐	☒	The exact sample size (*n*) for each experimental group/condition, given as a discrete number and unit of measurement
☐	☒	A statement on whether measurements were taken from distinct samples or whether the same sample was measured repeatedly
☒	☐	The statistical test(s) used AND whether they are one- or two-sided<br>*Only common tests should be described solely by name; describe more complex techniques in the Methods section.*
☒	☐	A description of all covariates tested
☒	☐	A description of any assumptions or corrections, such as tests of normality and adjustment for multiple comparisons
☐	☒	A full description of the statistical parameters including central tendency (e.g. means) or other basic estimates (e.g. regression coefficient) AND variation (e.g. standard deviation) or associated estimates of uncertainty (e.g. confidence intervals)
☒	☐	For null hypothesis testing, the test statistic (e.g. *F*, *t*, *r*) with confidence intervals, effect sizes, degrees of freedom and *P* value noted<br>*Give P values as exact values whenever suitable.*
☒	☐	For Bayesian analysis, information on the choice of priors and Markov chain Monte Carlo settings
☒	☐	For hierarchical and complex designs, identification of the appropriate level for tests and full reporting of outcomes
☒	☐	Estimates of effect sizes (e.g. Cohen's *d*, Pearson's *r*), indicating how they were calculated

Our web collection on statistics for biologists contains articles on many of the points above.

Software and code

Policy information about availability of computer code

Data collection	X-ray diffraction data were collected at the Stanford Synchroton Radiation Lightsource (SSRL) beamline 12-2 on a Pilatus 6M pixel detector (Dectris).<br><br>Data for single-particle cryo-EM were collected on either a Talos Artica or a Titan Krios transmission electron microscope, operating at 200 kV and 300 kV, respectively. For datasets collect on the Talos Artica, movies were recorded in super-resolution mode on a Falcon III camera (Thermo Fisher) at 1.436 Å•pixel-1 or a K3 camera (Gatan) at 0.4345 Å•pixel-1. Movies obtained from samples on the Titan Krios were collected in super-resolution mode on a K3 camera (Gatan) equipped with an BioQuantum energy filter (Gatan) with a 20 eV slit width at 0.4327 Å•pixel-1. EM data were collected using SerialEM software.
Data analysis	X-ray diffraction data from a single crystal were indexed and integrated in XDS and merged with AIMLESS in the CCP4 software suite. Structures were determined by molecular replacement in PHASER using coordinates of the BG24 Fab (PDB 7UCE), after removal of CDR loops and independent searches of the VHVL and CHCL domains. Models were refined using rigid body and B-factor refinement in Phenix, followed by several cycles of iterative manual building in Coot and real-space refinement with TLS groups in Phenix.<br><br>The cryo-EM data processing workflow was preformed similarly for all datasets using RELION. Movies were motion-corrected using MotionCor2 after binning and GCTF was used to estimate CTF. RELION was used for reference-free 2D-classifcation. An ab initio model was generated using cryoSPARC6using a subset of particles for each dataset and used as a reference in 3D classification in RELION. 3D classes representing a defined complex were selected for 3D auto-refinement and post processing in RELION.<br><br>Cryo-EM model coordinates were generated by fitting reference gp120 (PDB 5T3Z), gp41(PDB 5T3Z), 10-1074 (PDB 5T3Z), and BG24-derivitive

Fabs (this study) chains into cryo-EM density with UCSF Chimera. Initial models were refined using the Phenix command phenix.real_space_refine. Sequence updates to the model and further manual refinement was conducted with Coot. Iterative rounds of Phenix auto-refinement and manual refinements were done to generate the final models.

Structure figures were made using PyMol (Schrödinger LLC), UCSF Chimera, and UCSF ChimeraX. PyMol was used to calculate r.m.s.d. values after pairwise alignment of Cα atoms. PDBePISA was used to calculate buried surface areas.

For manuscripts utilizing custom algorithms or software that are central to the research but not yet described in published literature, software must be made available to editors and reviewers. We strongly encourage code deposition in a community repository (e.g. GitHub). See the Nature Portfolio guidelines for submitting code & software for further information.

Data

Policy information about availability of data

All manuscripts must include a data availability statement. This statement should provide the following information, where applicable:

- Accession codes, unique identifiers, or web links for publicly available datasets
- A description of any restrictions on data availability
- For clinical datasets or third party data, please ensure that the statement adheres to our policy

The atomic model generated for the X-ray crystallography structure of the BG24iGL-CDR3mat Fab in this study has been deposited in the Protein Data Bank (PDB) under accession code 7UGM. The cryo-EM maps and atomic structures have been deposited in the PDB and/or Electron Microscopy Data Bank (EMDB) under accession codes 7UGN and EMD-26490 for BG24iGL-CDR3iGL-GT1-10-1074 Class 1, EMD-26491 for BG24iGL-CDR3iGL-GT1-10-1074 Class 2, 7UGO and EMD-26492 for BG24iGL-CDR3mat-GT1-10-1074, 7UGP and EMD-26493 forBG24iGL-LC-GT1N276gp120-10-1074 Class 1, EMD-26494 for BG24iGL-LC-GT1N276gp120-10-1074 Class 2, EMD-26495 for BG24iGL-LC-GT1N276gp120-10-1074 Class 3, and 7UGQ and EMD-26496 for BG24CDRL-iGL-6405-10-1074. Local refinement maps used to model CDRL1s of BG24-derivatives have been deposited with PDB and EMDB accession codes for each respective structure.

Human research participants

Policy information about studies involving human research participants and Sex and Gender in Research.

Reporting on sex and gender	Not applicable.
Population characteristics	Not applicable.
Recruitment	Not applicable.
Ethics oversight	Not applicable.

Note that full information on the approval of the study protocol must also be provided in the manuscript.

Field-specific reporting

Please select the one below that is the best fit for your research. If you are not sure, read the appropriate sections before making your selection.

☒ Life sciences ☐ Behavioural & social sciences ☐ Ecological, evolutionary & environmental sciences

For a reference copy of the document with all sections, see nature.com/documents/nr-reporting-summary-flat.pdf

Life sciences study design

All studies must disclose on these points even when the disclosure is negative.

Sample size	Sample sizes and protein concentrations for X-ray crystallography and cryo-EM were estimated based on previous literatures and have been shown to be sufficient. Samples sizes for ELISA experiments were estimated for biological duplicates.
Data exclusions	Cyro-EM particle images with poor ice quality were excluded during data processing such as 2D classifications and 3D classifications to generate high-resolution EM maps, the method has been proven to be effective by numerous publications.
Replication	ELISA experiments were performed with n=2 independent biological replicates. Cryo-EM data were recorded and processed multiple times and showed same results with different resolutions. The datasets with the highest resolutions are reported here in this work.
Randomization	Randomization is not relevant to structural data or ELISAs.
Blinding	The same group of investigators designed and performed the experiments and analyzed the data for all results.

Reporting for specific materials, systems and methods

We require information from authors about some types of materials, experimental systems and methods used in many studies. Here, indicate whether each material, system or method listed is relevant to your study. If you are not sure if a list item applies to your research, read the appropriate section before selecting a response.

Materials & experimental systems

n/a	Involved in the study
☐	☒ Antibodies
☐	☒ Eukaryotic cell lines
☒	☐ Palaeontology and archaeology
☒	☐ Animals and other organisms
☒	☐ Clinical data
☒	☐ Dual use research of concern

Methods

n/a	Involved in the study
☒	☐ ChIP-seq
☒	☐ Flow cytometry
☒	☐ MRI-based neuroimaging

Antibodies

Antibodies used

Antibodies used in this study include: BG24-iGL-CDR3mat (this study), BG24-iGL-CDR3iGL (this study), BG24-CDRL1iGL (this study), BG24-LC-iGL (this study), BG24, PCIN63-71I1a, PCIN63-LC-UCA (this study), PCIN63-UCA, VRC01, VRC01-LC-iGL (this study), VRC01-iGL, N6, N6-LC-iGL (this study), N6-iGL, IOMA, IOMA-LC-iGL (this study), IOMA-iGL, CH103, CH106, CH103-UCA, PGT145.

For ELISA data, goat anti-human IgG Fc conjugated to horse-radish peroxidase (Southern BioTech) was used. Details of usage were described in the Methods section of this manuscript.

Validation

The structural binding mechanisms and binding affinities or HIV-1 Env-targeting antibodies have been described in previous literature:

Barnes, C. O. et al. A naturally arising broad and potent CD4-binding site antibody with low somatic mutation. Sci. Adv. 8, eabp8155 (2022).

Umotoy, J. et al. Rapid and Focused Maturation of a VRC01-Class HIV Broadly Neutralizing Antibody Lineage Involves Both Binding and Accommodation of the N276-Glycan. Immunity 51, 141-154.e6 (2019).

Zhou, T. Structural Basis for Broad and Potent Neutralization of HIV-1 by Antibody VRC01. 1–8 (2010).

Huang, J. et al. Identification of a CD4-Binding-Site Antibody to HIV that Evolved Near-Pan Neutralization Breadth. Immunity 45, 1108–1121 (2016).

Gristick, H. B. et al. Natively glycosylated HIV-1 Env structure reveals new mode for antibody recognition of the CD4-binding site. Nat. Publ. Group 23, 906–915 (2016).

Liao, H.-X. et al. Co-evolution of a broadly neutralizing HIV-1 antibody and founder virus. Nature 496, 469–476 (2013).

Cupo, A. et al. Optimizing the production and affinity purification of HIV-1 envelope glycoprotein SOSIP trimers from transiently transfected CHO cells. PLOS ONE 14, e0215106 (2019).

The binding specificity for goat anti-human IgG Fc conjugated to horse-radish peroxidase has been validated through a commercial source (https://www.southernbiotech.com/goat-anti-human-igg-hrp-2040-05). The effectiveness of this antibody has been repeatedly tested in ELISA experiments in our lab.

Eukaryotic cell lines

Policy information about cell lines and Sex and Gender in Research

Cell line source(s)	Expi293F cells (Thermo Fisher)
Authentication	Cell lines were not authenticated in the lab.
Mycoplasma contamination	No contamination.
Commonly misidentified lines (See ICLAC register)	No commonly misidentified lines were used.
